# Energy-Related Scatter Analysis for Determining the Effective Point of Measurement of Cylindrical Ion Chamber in Heavy Charged Particle Carbon Ion Beam

**DOI:** 10.1155/2021/8808537

**Published:** 2021-10-22

**Authors:** Xiao-Yun Ma, Yan-Shan Zhang, Wan-Bin Meng, Yin Qi, Qiang Li, Yan-Cheng Ye, Jia-Ming Wu

**Affiliations:** ^1^Department of Heavy Ion Center of Wuwei Cancer Hospital, Gansu Wuwei Academy of Medical Sciences, Gansu Wuwei Tumor Hospital, Wuwei City, Gansu Province 733000, China; ^2^Institute of Modern Physics, Chinese Academy of Sciences, 509 Nanchang Road, Lanzhou 730000, China; ^3^Department of Medical Physics, Chengde Medical University, Chengde City, Hebei Province 067000, China; ^4^Department of Radiation Oncology, Yee Zen General Hospital, Tao Yuan City 398, Taiwan

## Abstract

**Purpose:**

An experimental and mathematical study for determining the effective point of measurement (*P*_eff_) for a Farmer-type cylindrical chamber in a carbon ion passive scatter beam is presented.

**Methods:**

The ionization depth curves measured by the Bragg peak chamber were plotted according to the position of the inner surface of the entrance window, while the Farmer chamber was plotted at the tip of the cylindrical geometric center. The ionization depth curves measured by a cylindrical chamber in the 3D water phantom were then compared with a high-precision parallel-plate PTW Bragg peak chamber for inspecting the upstream shift correction of the cylindrical chamber in the carbon ion beam. A component of the vertical and horizontal integration method and the barrier model, cos*φ* = 1 − [2*αR*_*L*_/(1 + *α* − *R*_*L*_)], for analyzing the shift of effective point of measurement in different carbon ion energies and various field sizes, were studied.

**Results:**

The shift between the maximum peak of the Bragg peak chamber and the Farmer chamber in a field size of 10 cm × 10 cm with an energy of 330 MeV/u of carbon ion is 2.3 mm. This upstream shift corresponds to (0.744 ± 0.07)*r*, where *r* is the Farmer chamber inner radius of 3.05 mm. Carbon ion energy from 120 MeV/u to 400 MeV/u with different field sizes show different shifts of effective point of measurement in a range of (0.649 ± 0.02)*r* to (0.843 ± 0.06)*r* of 3 cm × 3 cm at an energy of 400 MeV/u and 10 cm × 10 cm at an energy of 120 MeV/u, respectively. The vertical and horizontal scatter analysis by the barrier model can precisely describe the shift of the effective point of measurement at different carbon ion energies with various field sizes.

**Conclusions:**

We conclude that the Farmer chamber can be used for a patient-specific dose verification check in carbon ion beam treatment if *P*_eff_ is well calibrated.

## 1. Introduction

The Institute of Modern Physics (IMP), affiliated with the Chinese Academy of Sciences (CAS), was founded in 1957 in Lanzhou, China. To take the advantage of full usage of the research facilities at IMP, the National Laboratory of Heavy Ion Accelerator, Lanzhou (NLHIAL), was established at IMP in 1991 [1]. Our *W*uWei *H*eavy *I*on *C*enter, Wuwei *C*ancer *H*ospital, Gansu, China (*WHICH*) heavy-ion facility established by IMP, CAS at 2014, was the first generation commercialized product transformed from a laboratory-based cancer treatment facility in China.

The protocol currently used to determine the effective point of measurement (*P*_eff_) for electron therapy at different beam qualities was the provenance of Dutreix [2, 3] proposing the effective point of measurement upstream from the chamber center with a certain ratio of cavity radius *r* for a cylindrical chamber. The shift in the point of measurement takes place because of the cylindricality of the chamber cavity [4]. When cylindrical chambers are used for electron measurement with the chamber geometric central axis aligned to the isocenter, electrons are focused by the chamber cylindricality causing denser electron clouds upstream from the center of the chamber. It does also present a problem in the carbon ion beam measurement by using the Farmer chamber because the fluency inside the cavity has higher average energy than at the same point in the water since carbon ion beams lose less energy in the cavity's air. If there is a gradient of carbon ion beam fluence across the cavity, a displacement in the point of measurement is needed to the dose measured at a point corresponding to the center of the chamber [5]. The perturbation effect is much more complicated for carbon ion beam measurement using a cylindrical chamber. The shape of the carbon ion beam fluency spectrum is continuously changing with depth in the chamber cavity. [6–8].

As for plane-parallel chambers, since the front plane (toward the source) of the air cavity is flat and is exposed to a uniform fluency of the charged particles, the point of measurement is at the front surface of the cavity. If the plane-parallel chamber has a small plate separation and the charged particle fluency is mostly forward-directed, it is reasonable to assume that the point of measurement is the front surface of the cavity.

The absolute dose of QA check or patient-specific dose verification is usually conducted by a cylindrical chamber instead of the parallel-plate chamber (PP chamber) due to the uncertainty perturbation calibration under Co-60 of the PP chamber. However, the plane-parallel chamber (sometimes also called parallel-plate chambers) is the “golden standard” in the Bragg peak measurement because the electrode spacing of the plane-parallel chambers is small (~1 mm). The thin wall or window of the parallel-plate chamber allows measurements practically at the water phantom surface without significant wall attenuation, and a perturbation of the half-cylindrical structure protrudes above the water surface. However, parallel-plate chambers are designed for radiation measurement with the chamber facing vertically to the beam central axis. The parallel-plate chamber lacks side scatter and backscatter fully accumulating functions; therefore, parallel-plate chambers are not suitable for absolute dose measurement.

The Farmer chamber, thereafter, becomes widely used in the absolute dose measurement of photons, electrons, protons, and heavy charged particles.

Some of the investigators presented their study only on a fixed carbon or proton energy, and lack of the offset correction varies with beam energy in the effective point of measurement.

According to the characteristics of the plane-parallel chamber, this study was based on the comparison of relative ionization depth curves measured by a cylindrical chamber and with a plane-parallel Bragg peak chamber to see the displacement of the effective point of measurement in the carbon ion beam. The vertical and horizontal scatter analysis by the Barrier model was then introduced to analyze the shift of the effective point of measurement at different energies and various field sizes in the carbon ion beam.

## 2. Materials and Methods

### 2.1. Introduction of Institute and Dose Delivery System

Our facility was a modification from the prototype of the Heavy Ion Research Facility in Lanzhou (HIRFL) and started to treat patients in earlier 2020. Our WHICH consists of an ECR ion source, an injection cyclotron SFC (energy constant *K* = 69), a cyclotron SSC (energy constant *K* = 450) as an injector offering charged particles to the main synchrotron ring to accelerate sufficient particle energy, and flux for use in four treatment rooms:

- Room 1: horizontal nozzle alone with a scanning beam
- Room 2: horizontal + vertical nozzles with a passive scatter beam
- Room 3: vertical nozzle alone with a scanning beam
- Room 4: 45° nozzle alone with a passive scatter beam for treatment of cancer patients

At WHICH, the five carbon ion beam energies are 120 MeV/u, 190 MeV/u, 260 MeV/u, 330 MeV/u, and 400 MeV/u, and all of them are equipped with maximum field sizes of 12 cm × 12 cm and 22 cm × 22 cm in the passive scatter beam and the pencil scanning beam, respectively. The cylindrical ionization chambers are used for the daily calibration of the beam quality assurance and the patient-specific dosimetric verification measurement at the middle of the Spread Out of Bragg Peak (SOBP) in a solid water phantom for comparison with the dose calculated by our home-made ciPlan (carbon ion beam Planning) treatment planning system.

The measurements of *P*_eff_ were carried out at WHICH in five carbon ion energies of 120 MeV/u, 190 MeV/u, 260 MeV/u, 330 MeV/u, and 400 MeV/u, and each energy value has four field sizes of 3 × 3 cm^2^, 5 × 5 cm^2^, 8 × 8 cm^2^, an 10 × 10 cm^2^ in this study. Since the plane-parallel chamber has a small plate separation and the charged particle fluency is mostly forward-directed, it is explicit that the point of measurement is the front surface of the cavity. The ionization depth curve measured by the plane-parallel chamber can be treated as the golden standard curve, thus the ionization depth curve measured by the Farmer chamber can then be compared with the standard curve to derive *P*_eff_.

### 2.2. Description of Ionization Chamber and Phantom

The PTW Bragg peak chamber type 34080 and type 34070 (PTW-Freiburg, Germany) were used for ionization depth curve measurement as a reference and in field detectors, respectively. The PTW Farmer chamber type 30013 was adopted for the displacement shift investigation experiment.  Table 1 lists these chambers' geometric and technical parameters in detail to calculate the water equivalent thickness of the chamber measurement point in PTW 3 D water phantom (PTW Freiburg, T41029-00006).

**Figure 1:**
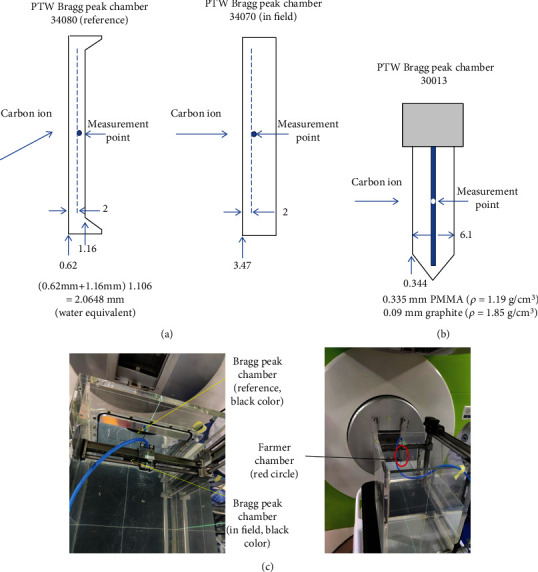
The parameters for water equivalent thickness calculation and the point of measurement of the Bragg peak chamber 34080 (reference), the Bragg peak chamber 34070 (in field) (a), and the Farmer 30013 chamber (b). The reference chamber is set up outside the water phantom, while the in-field and Farmer chambers are embedded in water when measured (c).

The golden Bragg curve was measured with the in-field Bragg peak chamber owing to the advantage of the large sensitive area in large-area ionization chambers. The in-field chamber was held by a chamber holder on a T bar driven by a high-precision stepping motor (10 *μ*m per step) to adjust the chamber measurement position in the water tank (Figure 1(a)).

Table 1 shows the chamber's geometric and technical parameters for calculating the water equivalent thickness of the chamber measurement point in the water phantom.

The calculation of the water equivalent thickness of the chamber wall for the PTW Bragg peak chamber type 34080 (reference) is 2.0648 mm. The entrance window for the PTW Bragg peak chamber type 34070 (in field) is 0.1 mm lacquer, 3.35 mm PMMA, and 0.02 mm graphite. The total physical dimension is 3.47 mm, and the water equivalent thickness calculation for type 34070 is 4.022 mm (which is calculated as 3.35 mm × 1.16 + 0.02 mm × 1.85 × (1.16/1.19) + 0.1 mm). The wall materials of the PTW Farmer chamber type 30013 are 0.335 mm PMMA (*ρ* = 1.19 g/cm^3^) and 0.09 mm graphite (*ρ* = 1.85 g/cm^3^), and the water equivalent thickness calculation for PTW 30013 is 4.022 mm (for details, please refer to Figure 1)

### 2.3. Measurement of Ionization Depth Curves by the Bragg Peak Parallel Plate Ion Chamber and by the Farmer Cylindrical Chamber

The point of measurement was inside of the entrance window, at the front surface of the cavity center of the Bragg peak chamber type 34070.

The effective point of measurement was conducted with the PTW Farmer chamber type 30013 embedded in water in the water tank (Figure 1(a). The point of measurement was conducted on the detector axis, 13 mm behind the tip of the Farmer chamber type 30013 (Figure 1(b)) and the setup of two chambers in the water phantom (Figure 1(c)).

The distance from the tank inner wall to the frontal surface of the PTW Bragg peak chamber type 34070 and the tip of the PTW Farmer chamber type 30013 was 7 mm and 25 mm, respectively. Before the experiment, a 3 cm × 3 cm carbon ion beam profile was measured at the proximal and distal points on the plateau range of the ionization depth curve before the Bragg peak to check if the chamber measurement point movement tracks were always on the center of the field, in other words, on the beam's central axis. The divergence of the beam is below 0.1°, so that the path length in the water was equal for all points in the active volume of the chambers.

### 2.4. Depth Dose Curve Derivation from Ionization Depth Curve of a Farmer Chamber

Our calibration procedure and dosimetric protocol for determining the absorbed dose in heavy charged particles followed what was proposed by Hartmann in 1999 et al. [9]. A daily dose measurement with a Farmer chamber in a phantom with a homogeneous monoenergetic field at five different carbon ion energies is used to calibrate the monitors in terms of absolute particle numbers. This measurement is performed in the plateau region of the depth dose at a depth of 2.1 mm. The calibration factor *K*(*E*), defined as the number of particles *N* per monitor unit MU, is given by

(1) $$\begin{array}{c} K\left(E\right)=\frac{N}{\text{MU}}=\frac{D_{\text{meas}}}{S_{\left(E\right)X}\text{MU}}A, \end{array}$$

where *D*_meas_ is the dose measured in a phantom, *S*_(*E*)*X*_ is the mass stopping power of carbon ions with the initial energy *E* corrected for the depth of measurement *x*, and *A* is the area that is homogeneously irradiated with a predefined number of monitor units.

The derivation from the ionization depth curve to the depth dose curve of the Farmer chamber has followed the protocol of protons described by Vynckier et al. [10] using a Co-60 exposure calibration factor, described as

(2) $$\begin{array}{c} D_{w\left(\text{Peff}\right)}=M_{\text{corr}}N_{w,\text{Co}‐60}K_{Q}. \end{array}$$

*M*_corr_ is the measured charge in the air cavity corrected for deviations from the laboratory temperature and air pressure conditions. *N*_*w*,Co‐60_ is the absorbed dose to water calibration factor for Co-60 gamma radiation under laboratory temperature and air pressure conditions. *K*_*Q*_ denotes the beam quality of carbon to Co-60.

(3) $$\begin{array}{c} K_{Q}=\frac{{\left(w/e\right)}^{\text{C}‐12}S_{a}^{w}}{{\left(w/e\right)}^{\text{C}0‐60}{\left(L/\rho \right)}_{a}^{w}\;}. \end{array}$$

The data of the stopping power ratio for carbon ions are from Hiraoka et al. [11].

(*w*/*e*)^Co−60^ is the *w* value, (*L*/*ρ*) is the ratio of the restricted linear energy transfer for water to air using the Spencer–Attix cavity theory [12], and (*w*/*e*)^C−12^ is the effective *w* value for the carbon ions including all fragments.

### 2.5. The Comparison of Depth Dose Curve Converted via the Farmer Chamber Ionization Curve with GAF EBT 3 Film

We used Gafchromic EBT3 films (Ashland Specialty Ingredients GP, NJ USA; Lot # 04022001) for the depth dose curve measurement for determining the effective point of a measurement experiment. The film processing and dose profile measurements followed international protocols [13]. A preexposure technique was used for the calibration curve derivation [14]. This was performed by giving each film a priming dose of 2 Gy to homogenize the film density using our WHICH facility with a dose of 1 Gy at the carbon ion energy of 330 MeV/u. We then measured the dose homogeneity using a densitometer. Graded doses of 5, 10, 15, 40, 60, 80, 100, 150, and 200 cGy were given to the GAF chromic film to obtain the Hurter-Driffield calibration curve (H-D curve).

All exposed films of the depth dose curve were then scanned with an Epson Expression 11000XL scanner in the 48-bit RGB mode (16 bits per color), and the data were saved in tagged image file format (TIFF) and analyzed by the VeriSoft imaging procession software. A red filter was placed on top of the GAF films before scanning to increase the slope of the H-D curve, thereby raising the resolution of the dose-OD curves [15].

The depth dose curve derived from the ionization depth curve from the Farmer chamber was then compared not only by the Bragg peak parallel-plate chamber but also with the depth dose curves measured by GAF Chromic EBT3 films.

### 2.6. The Calculation of the Effective Point of Measurement for the Cylindrical Chamber by Dutreix

The calculation process of the Dutreix method has the same definition as a cross-section of a cylindrical chamber that is exposed to a parallel, uniform, and forwardly directed fluence *Φ* of electrons. The effective point of measurement, *y*_eff_, therefore, can be determined by weighting the displacement *y* by the number of electrons (*Φ*∙*ds*∙cos(*θ*)) entering the chamber and the track length of 2*y*. To be noticed, the *θ* angle starts from the vertical axis, and the *dθ* is the differential angle of *θ* in Appendix A. 2y∙ds∙cos(*θ*) means the column receives electrons entering from the chamber surface.

*y*_eff_ is defined by Dutreix as follows:

(4) $$\begin{array}{c} y_{\text{eff}}=\frac{\int_{0}^{\left(\pi /2\right)}y∙2y∙ds\cos\left(\theta \right)∙\Phi }{\int_{0}^{\left(\pi /2\right)}2y∙ds\cos\left(\theta \right)∙\Phi }, \end{array}$$

where*ds* = (*dθ*/2*π*)∙2*πr* = *rdθ*,*y* = *r*cos(*θ*),*x* = *r*sin(*θ*).

The numerator is as follows:

(5) $$\begin{array}{c} \int_{0}^{\left(\pi /2\right)}y∙2y∙ds\cos\left(\theta \right)=\int_{0}^{\left(\pi /2\right)}r\cos\left(\theta \right)∙2r\cos\left(\theta \right)∙rd\theta ∙\cos\left(\theta \right)=2r^{3}\int_{0}^{\left(\pi /2\right)}\cos^{3}\left(\theta \right)d\theta . \end{array}$$

The denominator is as follows:

(6) $$\begin{array}{c} \int_{0}^{\left(\pi /2\right)}2y∙ds\cos\left(\theta \right)=2r^{2}\int_{0}^{\left(\pi /2\right)}\cos^{2}\left(\theta \right)d\theta . \end{array}$$

Therefore, *y*_eff_ is as follows:

(7) $$\begin{array}{c} y_{\text{eff}}=\frac{\left(4/3\right)r^{3}}{2r^{2}∙\left(\pi /4\right)}. \end{array}$$

For a detailed derivation, please refer to Appendix A.

It is reasonable that the induced electrons of carbon ion beams are more condensed than the electrons produced by the linear accelerator in the Dutreix study. In addition, the different contributions of the high and low energies of the carbon ion beam's side scatter to the vertical and horizontal axes reveal the importance of separate calculations for the low and high carbon ion beam energies of their scatter contribution on the horizontal and vertical axes in determination of the effective measurement point for a cylindrical ion chamber.

### 2.7. Scatter Analysis by Vertical and Horizontal Components to Determine the Effective Point of Measurement of the Farmer Chamber in Different Carbon Ion Beam Energy

The carbon ion beam spectrum in the cavity is different from that at depth in the phantom without the presence of the chamber. If a detailed knowledge of the spectrum in the cavity and in the phantom is well known, then it will be a useful way to study the perturbation effect of the cylindrical chamber cavity in carbon ion beams. Therefore, scatter analysis for calculating the effective point of measurement inside a cylindrical ion chamber needs to be clarified in carbon ion beams.

**Figure 2:**
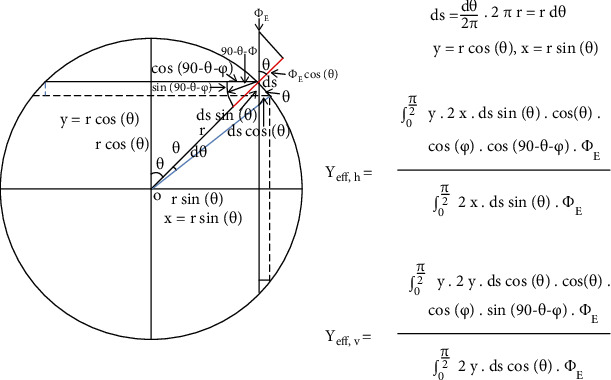
A two-scatter method for calculating the contribution of carbon ion beam scatter components on the vertical and horizontal axes for the effective point of measurement of cylindrical ion chambers on carbon ion beam measurement.

The calculation of the carbon ion beam scatter contribution of vertical and horizontal components inside the cylindrical chamber was calculated by the spreading angle of normal incident carbon ion beams in Figure 2.

The calculation of scattering contribution of the charged particles for effective point of measurement calculation to the *x*-axis on the horizontal direction is described as follows:

(8) $$\begin{array}{c} y_{\text{eff},\text{h}}=\frac{\int_{0}^{\left(\pi /2\right)}y∙2x∙ds\sin\left(\theta \right)∙\cos\left(\theta \right)∙\cos\left(\varphi \right)∙\cos\left(90-\theta -\varphi \right)∙\Phi E}{\int_{0}^{\left(\pi /2\right)}2x∙ds\sin\left(\theta \right)∙\Phi E}, \end{array}$$

where *y*_eff,*h*_ denotes the carbon ion beam scatter contribution of the charged particles for effective point of measurement calculation to the *x*-axis on the horizontal direction.

The calculation of the carbon ion beam scatter contribution for effective point calculation to the *y*-axis on the vertical direction is described as follows:

(9) $$\begin{array}{c} y_{\text{eff},\text{v}}=\frac{\int_{0}^{\left(\pi /2\right)}y∙2y∙ds\cos\left(\theta \right)∙\cos\left(\theta \right)∙\cos\left(\varphi \right)∙\sin\left(90-\theta -\varphi \right)∙\Phi E}{\int_{0}^{\left(\pi /2\right)}2y∙ds\cos\left(\theta \right)∙\Phi E}, \end{array}$$

where *y*_eff,*v*_ denotes the carbon ion beam scatter contribution of the charged particles for effective point of measurement calculation to the *y*-axis on the horizontal direction.

Please refer to Appendix B for details of the calculation process.

### 2.8. The Carbon Ion Beam Scatter Angle Analysis for Calculating the Effective Measurement Point in Cylindrical Ion Chamber

A modified analytical approach to provide an estimate of beam spreading for a carbon ion beam incident normal to a medium was adopted in the scatter analysis of a charged particle beam incident to a cylindrical chamber. The modified analysis barrier model was originated by the Rutherford model [16]:

(10) $$\begin{array}{c} \cos\varphi =1-\left[\frac{2\alpha R_{L}}{1+\alpha -R_{L}}\right], \end{array}$$

where *φ* is the spreading angle of a normal incident charged particle beam.

The screening factor *α* has been given as follows:

(11) $$\begin{array}{c} \alpha =\frac{\varepsilon e^{-1.1}{\text{Z}}^{-0.68}}{\text{Ec}}, \end{array}$$

where *ε* is the barrier ratio for different carbon ion energies with various field sizes, *Z* is the atomic number, Ec is the carbon ion energy in MeV.

*R*_*L*_ is a parameter for describing Gaussian distribution in a statistical distribution.

## 3. Results

The ionization depth curves measured by the Bragg peak chamber and the Farmer chamber were carried out for 3 cm × 3 cm, 5 cm × 5 cm, 8 cm × 8 cm, and 10 cm × 10 cm with 120 MeV/u, 190 MeV/u, 260 MeV/u, 330 MeV/u, and 400 MeV/u carbon ion beams. The effective point of measurement upstream shift ratio is listed in Table 2. The smallest shift ratio at a small field size of 3 cm × 3 cm with an energy of 120 MeV/u is 0.782*r*, while the largest shift ratio at a large field size of 10 cm × 10 cm with an of energy 400 MeV/u is 0.723*r*.

Table 2 shows the effective point of measurement shift ratio, which means an upstream shift to the chamber radius *r* ratio. *R* is the inner radius of the Farmer chamber, which is 3.05 mm. The negative sign denotes that the effective point of measurement is upstream toward the source.

**Figure 3:**
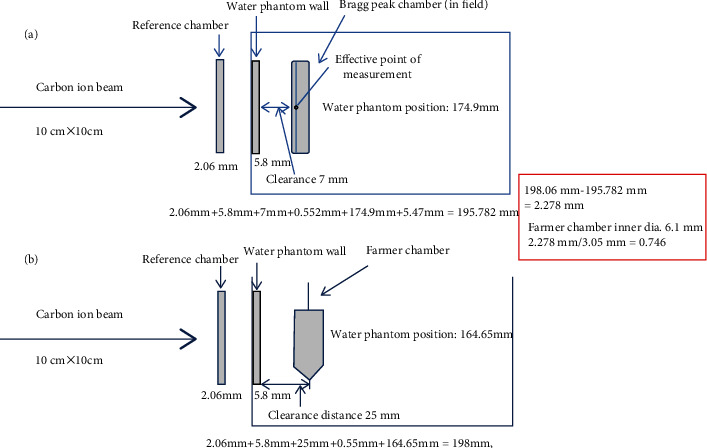
Schematic set-up of the effective point of measurement using the Bragg peak chamber and the Farmer ion chamber in the carbon ion beam. The water equivalent thickness for the carbon ion beam through the Bragg peak reference chamber, water tank wall, and the clearance of anticollision distance to the Bragg peak in the field chamber item by item is calculated (a), while the water equivalent thickness for the carbon ion beam through the Bragg peak reference chamber, water tank wall, and the clearance of anticollision distance to the Farmer chamber item by item is calculated (b).

**Figure 4:**
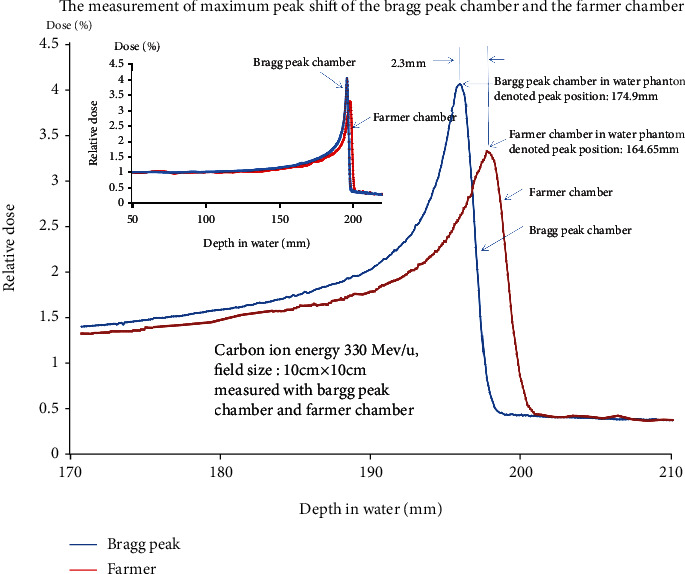
One of the ionization depth curves of the Bragg peak shift measured by the Bragg peak chamber 34070 and the Farmer chamber 30013 for 330 MeV/u at a field size of 10 cm × 10 cm. The measurement point position in the water phantom at the peak of the ionization depth curve of the Bragg peak chamber is 174.9 mm, while for the Farmer chamber, it is 164.65 mm. For a detailed calculation of the total water equivalent thickness of the BP chamber and the Farmer chamber, please refer to Figure 3.

According to Figure 3, the water equivalent thickness for a carbon ion beam through the Bragg peak reference chamber, water tank wall, and the clearance of anticollision distance to the Bragg peak in the field chamber item by item is calculated to be 195.782 mm (Figure 3(a)), While the water equivalent thickness for a carbon ion beam through the Bragg peak reference chamber, water tank wall, and the clearance of anticollision distance to the Farmer chamber item by item is calculated to be 198.06 mm (Figure 3(b)). The deviation between 198.06 mm and 195.782 mm is 2.278 mm, which is equal to 0.746*r*, where *r* is the Farmer chamber inner radius. One of the examples of ionization depth curves of the Bragg peak shift measured by the Bragg peak chamber and the Farmer chamber for 330 MeV/u at a field size of 10 cm × 10 cm is in Figure 4. The denoted peak position (chamber set-up position in a water phantom) of the Bragg peak chamber in a water phantom is 174.9 mm, and the denoted peak position of the Farmer chamber in a water phantom is 164.65 mm.

The analysis of the effective point of measurement by the barrier model is in Table 3. Table 3 shows only the smallest and largest field sizes of 3 cm × 3 cm and 10 cm × 10 cm, respectively. The parameters in use of *R*_*L*_, *Z*, *E*_*c*_, and FS are -0.6, 7.4, carbon ion energy, and field size irradiated from the nozzle, respectively.

The calculation of effective measurement that is shown in Table 3 should be separated into two components, horizontal and vertical contributions from the scatter charged particle inside the chamber cavity for calculating the effective point of measurement by using the barrier model in a cylindrical ion chamber.

For the best fit to link the carbon ion beam energy and the field size used for this model in the measurement, we have the following:

(12) $$\begin{array}{c} \varepsilon =1.6\frac{e^{\ln\left(Ec\right)}}{{FS}^{0.5}}. \end{array}$$

The comparison of depth dose curves between the Farmer chamber and GAF Chromic EBT3 reveals a good agreement with the position deviation within 0.1 mm.

## 4. Discussion

### 4.1. Uncertainties in the Ionization Depth Curve Measurements

The errors in the wall thickness to water equivalent derivation of the phantom, chamber measurement position alignment uncertainty, the determination of the offset for the mounted chambers, and the accuracy of the motor-driven water phantom mechanical device cause the overall uncertainty of the measurement. A 3 cm × 3 cm profile measurement at proximal and distal points in the plateau range ensures that our alignment keeps within 0.1 mm in 100 mm to an increasing error with depth in water. The entrance window of the Bragg peak type 34080 reference chamber is made of PMMA with a wall thickness of 0.62 mm plus electrode material PMMA with a graphite coat of 1.16 mm which corresponds to 2.0648 mm of water, where a conversion factor for PMMA to a water equivalent path length of 1.16 was used. This factor has an uncertainty of 3% which results in uncertainty for a depth of 0.1 mm.

The accuracy of the positioning of the chambers within the phantom is specified by the manufacturer to be 0.1 mm. The offset of the distance between the entrance wall and the chambers mounted to the phantom was measured mechanically with an accuracy of 0.1 mm.

The accuracy of the alignment of the measurement position was also checked with the aid of lasers to keep the setup error within 0.1 mm. At least two measurements have been performed for each chamber position.

The first point of measurement at the smallest depth was repeated after the measurement at the deepest point to check the overall accuracy of reproducibility. The dosimeter introduces an uncertainty in the charge measurement of 0.5% according to the manufacturer.

The quenching effect in EBT film was found due to the high LET in the carbon ion beam when the PDD measurement was conducted. In the plateau region of the Bragg curve of the film, a response can be well described by a simple polynomial function because the response is almost linear. In regions covering the Bragg maximum peak, the film response suffers a quenching and the optical density values were found to be 20–30% lower than expected. However, there are doses that are underestimated, but the position of the Bragg peak remains constant reflecting the peak where different carbon energies should impart.

### 4.2. The Scatter Contribution of Vertical and Horizontal Components of Carbon Ion Beam Inside the Cylindrical Chamber

**Figure 5:**
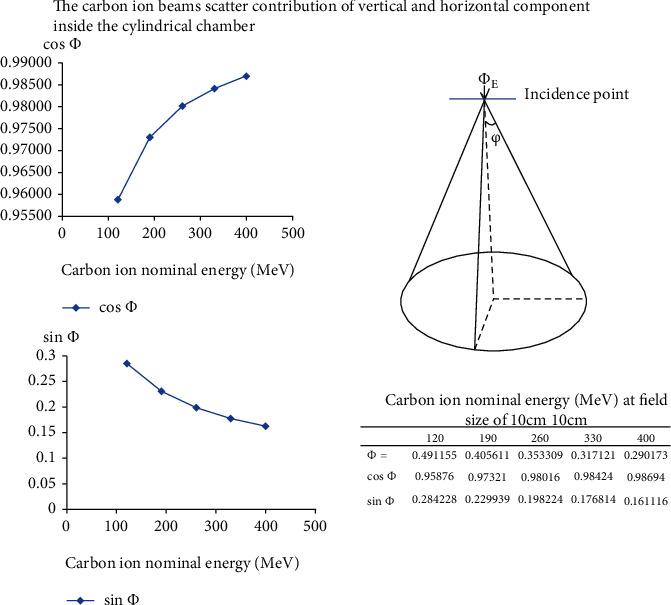
The spreading angle *φ* decreases when incident carbon ion beam energy increases. The vertical component (cos(*φ*)) of carbon ion beams with energy fluency *Φ*_*E*_ inside the cylindrical chamber cavity increases when the spreading angle *φ* decreases.

The spreading angle *φ* decreases when incident carbon ion beam energy increases (Figure 5). The vertical component (cos(*φ*)) of carbon ion beams with energy fluence *Φ*_*E*_ inside the cylindrical chamber cavity increases when the spreading angle *φ* decreases.

The percent depth dose curves measured by the cylindrical chamber were compared to the results measured by parallel plate chamber. The effective point of measurement for the cylindrical chamber was then shifted to match the ionization depth dose curves measured by the parallel plate chamber since in the parallel plate chambers, there are effective points of measurement within the air-filled cavity and that the resulting depth dependence of the overall perturbation correction is largely depth independent. The shift quantities of chamber inner radius *r* for the cylindrical chamber to match the ionization depth dose curves measured by plate-parallel chamber are shown in Table 2.

The analysis of an effective point shift for the Farmer chamber calculated by the barrier model is listed in Table 3. The side-scattering effect is stronger for the higher-energy carbon ion beams than the lower-energy carbon ion beams when the medium is replaced by the chamber air cavity so the effective point of measurement is affected by more contribution from the horizontal than the vertical direction compared to that of low-energy carbon ion beams. On the contrary, the forward scattering effect is stronger for higher-energy carbon ion beams when the medium was replaced by the chamber air cavity so the effective point of measurement is affected by more contribution from the vertical than the horizontal direction compared to that of low-energy carbon ion beams. Furthermore, more electrons ejected by higher-energy carbon ion beams focusing a denser electron cloud on the inner surface of the cylindrical air cavity lead to the effective point of measurement of a larger upstream shift to source than the lower energy carbon ion beams do.

The deviation of the upstream shift ratio to the inner radius of the Farmer chamber calculated by the barrier model compared to the Bragg peak measured by the plate-parallel Bragg peak chamber is about -2.8% for a carbon ion energy of 120 MeV/u at the smallest field size of 3 cm × 3 cm. Since the Bragg peak for carbon ion energy at 120 MeV/u is only 16.1 mm, and the inner radius of the Farmer chamber is 3.05 mm, it is reasonable to doubt the setup accuracy, and lack of electron equivalent increases the measurement uncertainty of the effective point of measurement of the Farmer chamber. The deviations for the rest energy values of 190 MeV/u, 260 MeV/u, 330 MeV/u, and 400 MeV/u are 0.1%, 1.7%, -2.5%, and -1.3%, respectively, at the smallest field size of 3 cm × 3 cm according to Tables 2 and 3.

The deviations of the upstream shift ratio to the inner radius of the Farmer chamber predicted by the barrier model compared to the Bragg peak measured by the plate-parallel Bragg peak chamber for a carbon ion energy of 120 MeV/u but at field sizes of 5 cm × 5 cm, 8 cm × 8 cm, and 10 cm × 10 cm are -1.7%, -1.5%, and -0.6%, respectively (not listed in Tables 2 and 3).

According to Tables 2 and 3, the deviations of the upstream shift ratio to the inner radius of the Farmer chamber analyzed by the barrier model compared to the Bragg peak measured by the plate-parallel Bragg peak chamber for carbon ion energies of 120 MeV/u, 190 MeV/u, 260 MeV/u, 330 MeV/u, and 400 MeV/u at a field size of 10 cm × 10 cm are -1.1%, -1.2%, -0.4%, -0.5%, and -0.4%, respectively.

The contribution of low-carbon ion beam energy strong side scatter to the horizontal axis reveals the importance of a separate calculation for low- and high-carbon ion beam energy on the effective measurement point for a cylindrical ion chamber. The parameters listed in Table 3 for the scatter angle calculation are used to calculate the *y*_eff,*v*_ and *y*_eff,*h*_ for the effective point of measurement of high- and low-carbon ion beam energies in a cylindrical ion chamber.

The barrier model has successfully proven that the effective point of measurement calculations for the Farmer chamber is affected by the scatter contributions of *y*_eff,*v*_ and *y*_eff,*h*_ at different energy values and various field sizes for a carbon ion beam in this study.

## 5. Conclusion

The effective point of a measurement shift correction of the cylindrical ion chambers of a passive scatter carbon ion beam was calculated mathematically with horizontal and vertical scatter contributions in this study, and the shift predicted by the barrier model agrees with the ionization depth curves measured by the plate-parallel Bragg peak chamber within the range in low- or high-energy carbon ion beams.

Our new mathematical method replaced the conventional 0.85*r* shift of the effective point of measurement in a cylindrical ion chamber for electron beam calculation. The method presented in this report is unambiguous and reliable for the estimation of the absolute dose in carbon ion beam dosimetry.

We successfully fit the measurement data by using a mathematical method empirically to know the energy-related scatter pattern in the chamber effective point measurement offset correction to predict the offset correction in scanning beam energy-related single-field uniform dose delivery.

Applying the proposed shift calculation method from this study would increase the accuracy of absolute output calibration in high- and low-energy pencil-scanning carbon ion beams. This is especially of great importance if the effectiveness of measurement in a cylindrical ion chamber calculated by similar Monte Carlo-based complicated algorithms is compared with the method proposed in this study for the determination of the chamber positioning, then, clinical carbon ion beam dosimetry will be simplified.

## Figures and Tables

**Figure 6:**
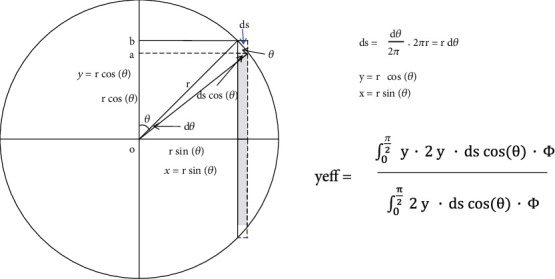
The derivation of the Dutreix 0.85*r* calculation with the *θ* angle starts from the vertical axis.

**Table 1:** Physical and material parameters of the Bragg peak chamber (reference/in field) and the Farmer chamber.

		Bragg peak chamber (reference) PTW 34080	Bragg peak chamber (in field) PTW 34070	Farmer chamber PTW 30013
Parameters	Active volume	10.5 cm^3^	10.5 cm^3^	0.6 cm^3^
	Chamber effective point of measurement	Inside of entrance window, center	Inside of entrance window, center	On detector axis, 13 mm behind the tip
Chamber wall	Inner diameter(mm)			6.1
	Length/thickness (mm)			0.335 mm PMMA (*ρ* = 1.19 g/cm^3^) 0.09 mm graphite (*ρ* = 1.85 g/cm^3^)
	Entrance window absolute thickness (mm)	0.1 mm lacquer	0.1 mm lacquer	
		0.5 mm PMMA	3.35 mm PMMA	
		0.02 mm graphite	0.02 mm graphite	
Water-equivalence		0.7 mm for photons	4.0 mm for photons	
Area density		72 mg/cm^2^	411 mg/cm^2^	Total 56.5 mg/cm^2^
Operating voltage (V)		400	400	400

**Table 2:** The effective point of measurement shift ratio (shift/radius) for the Farmer chamber in a carbon ion beam.

Carbon ion energy (MeV/u)					
Field size	Energy				
	120	190	260	330	400
3 × 3 cm^2^	−(0.782 ± 0.02)*r*	−(0.715 ± 0.03)*r*	−(0.678 ± 0.04)*r*	−(0.677 ± 0.05)*r*	−(0.649 ± 0.05)*r*
5 × 5 cm^2^	−(0.795 ± 0.03)*r*	−(0.746 ± 0.04)*r*	−(0.699 ± 0.05)*r*	−(0.694 ± 0.05)*r*	−(0.660 ± 0.06)*r*
8 × 8 cm^2^	−(0.811 ± 0.04)*r*	−(0.783 ± 0.04)*r*	−(0.725 ± 0.05)*r*	−(0.716 ± 0.06)*r*	−(0.695 ± 0.06)*r*
10 × 10 cm^2^	−(0.843 ± 0.05)*r*	−(0.825 ± 0.05)*r*	−(0.773 ± 0.06)*r*	−(0.744 ± 0.07)*r*	−(0.723 ± 0.06)*r*

(1) *r*: the inner radius of the Farmer chamber, 3.05 mm. (2) “-”: the negative sign “-” indicates that the effective point of measurement is upstream toward the source. (3) The Bragg peak position means a plate-parallel Bragg peak chamber measurement point position at 10 cm × 10 cm of different carbon energies in the water phantom.

**Table 3:** The prediction of an effective point shift of the Farmer chamber using the barrier model for field sizes 3 cm × 3 cm and 10 cm × 10 cm in different carbon ion energies.

	The energy of carbon ions (MeV/u)									
	120		190		260		330		400	
Field size (cm × cm)	3 × 3	10 × 10	3 × 3	10 × 10	3 × 3	10 × 10	3 × 3	10 × 10	3 × 3	10 × 10
*α* (screening factor)	0.84	0.19	0.53	0.12	0.39	0.09	0.31	0.07	0.25	0.06
cos *φ* = horizontal scatter cal.	0.71	0.88	0.77	0.92	0.81	0.94	0.84	0.95	0.86	0.96
2cos*Φ*sin*Φ*/15	0.07	0.05	0.07	0.05	0.06	0.04	0.06	0.04	0.06	0.04
2(cos*Φ*)^2^/15	0.07	0.10	0.08	0.11	0.09	0.12	0.09	0.12	0.10	0.12
Horizontal shift	0.55	0.40	0.43	0.33	0.37	0.25	0.30	0.20	0.30	0.17
Vertical scatter cal.										
8(cos*Φ*)^2^/15	0.27	0.42	0.32	0.45	0.35	0.47	0.37	0.48	0.39	0.49
cos*Φ*sin*Φ*/5	-0.10	-0.08	-0.10	-0.07	-0.10	-0.06	-0.09	-0.06	-0.09	-0.05
Vertical shift	0.21	0.43	0.28	0.48	0.32	0.52	0.36	0.54	0.34	0.55
Total shift (horizontal shift + vertical shift)	0.76	0.83	0.71	0.81	0.69	0.77	0.66	0.74	0.64	0.72

(1) The items 2cos*Φ*sin*Φ*/15 and 2(cos*Φ*)^2^/15 are substituted for horizontal scatter calculation in Appendix B. (2) The items 8(cos*Φ*)^2^/15 and cos*Φ*sin*Φ*/5 are substituted for vertical scatter calculation in Appendix B.

## Data Availability

The data used to support the findings of this study are available from the corresponding author upon request.

## Appendix

### A. The Derivation of the Dutreix 0.85*r* Calculation with the *θ* Angle Starts from the Vertical Axis (Figure 6)

(A.1)
$$\begin{array}{c} Y_{\text{eff}}=\frac{\int_{0}^{\left(\pi /2\right)}y∙2y∙ds\cos\left(\theta \right)∙\Phi }{\int_{0}^{\left(\pi /2\right)}2y∙ds\cos\left(\theta \right)∙\Phi },\\ ds=\frac{d\theta }{2\pi }.2\pi r=rd\theta ,\\ y=r\cos\left(\theta \right),\\ x=r\sin\left(\theta \right),\\ \int_{0}^{\left(\pi /2\right)}y∙2y∙ds\cos\left(\theta \right)=\int_{0}^{\left(\pi /2\right)}r\cos\left(\theta \right)∙2r\cos\left(\theta \right)∙rd\theta ∙\cos\left(\theta \right)=2r^{3}\int_{0}^{\left(\pi /2\right)}\cos^{3}\left(\theta \right)d\theta =2r^{3}\int_{0}^{\left(\pi /2\right)}\cos^{2}\left(\theta \right)d\sin\left(\theta \right)d\theta =2r^{3}\int_{0}^{\left(\pi /2\right)}\left(1-\sin^{2}\left(\theta \right)\right)d\sin\left(\theta \right)d\theta =2r^{3}\left(\left[\sin{\left.\left(\theta \right)\right|}_{0}^{\left(\pi /2\right)}\right]-\left[\frac{1}{3}\sin^{3}{\left.\left(\theta \right)\right|}_{0}^{\left(\pi /2\right)}\right]\right)=2r^{3}\left[\left(\sin\left(\frac{\pi }{2}\right)-\sin\left(\theta \right)\right)-\frac{1}{3}\sin^{3}\left(\frac{\pi }{2}\right)+\frac{1}{3}\sin^{3}\left(0\right)\right]=2r^{3}\left[\left(1-0\right)-\frac{1}{3}\;\left(1+0\right)\right]=\frac{4}{3}r^{3},\\ \int_{0}^{\left(\pi /2\right)}2y∙ds\cos\left(\theta \right)=\int_{0}^{\left(\pi /2\right)}2r\cos\left(\theta \right)∙rd\theta ∙\cos\left(\theta \right)=2r^{2}\int_{0}^{\left(\pi /2\right)}\cos^{2}\left(\theta \right)\text{d}\theta =2r^{2}\int_{0}^{\left(\pi /2\right)}\frac{1+\cos\left(2\theta \right)}{2}d\theta =2r^{2}\left[\int_{0}^{\left(\pi /2\right)}\left(\frac{1}{2}d\theta +\frac{1}{2}\cos\left(2\theta \right)d\theta \right)\right]=2r^{2}\left(\frac{1}{2}{\left.\theta \right|}_{0}^{\left(\pi /2\right)}+\frac{1}{4}\sin{\left.\left(2\theta \right)\right|}_{0}^{\left(\pi /2\right)}\right)=2{\text{r}}^{2}\left[\left(\frac{1}{2}.\frac{\pi }{2}-\frac{1}{2}.0\right)+\frac{1}{4}\left(\sin\left(\pi \right)-\sin\left(0\right)\right)\right]=2r^{2}.\frac{\pi }{4}\\ y_{\text{eff}}=\frac{\left(4/3\right)r^{3}}{2r^{2}.\left(\pi /4\right)}=\frac{8}{3\pi }r=0.85r. \end{array}$$

### B. The Derivation of the Vertical and Horizontal Components Generates Inside the Cylindrical Chamber for Carbon Ion Beam

(B.1)
$$\begin{array}{c} d\text{s}=\frac{d\theta }{2\pi }∙2\pi r=rd\theta ,\\ x=r\sin\left(\theta \right),\\ y=r\cos\left(\theta \right). \end{array}$$

*Φ*_*E*_ is the fluence of the charged particle. *Φ*_*E*_cos(*θ*) is the vertical component of the incidence charged particle with fluence *Φ*_*E*_ on the chamber cylindrical surface. *y*_eff,*h*_ denotes the charged particle scatter contribution for effective point calculation of the *x*-axis on the horizontal direction. *y*_eff,*v*_ denotes the charged particle scatter contribution for effective point calculation of the *y*-axis on the vertical direction.

The calculation of the charged particle's scatter contribution for effective point calculation of the *x*-axis on the horizontal direction is as follows:

(B.2) $$\begin{array}{c} y_{\text{eff},h}=\frac{\int_{0}^{\left(\pi /2\right)}y∙2x∙ds\sin\left(\theta \right)∙\cos\left(\theta \right)∙\cos\left(\varphi \right)∙\cos\left(90-\theta -\varphi \right)∙\Phi E}{\int_{0}^{\left(\pi /2\right)}2x∙ds\sin\left(\theta \right)∙\Phi \text{E}}. \end{array}$$

The numerator is as follows:

(B.3) $$\begin{array}{c} \int_{0}^{\left(\pi /2\right)}y∙2x∙ds\sin\left(\theta \right)∙\cos\left(\theta \right)∙\cos\left(\varphi \right)∙\cos\left(90-\theta -\varphi \right)∙\Phi E=2r^{3}\Phi_{E}\int_{0}^{\left(\pi /2\right)}\cos\left(\theta \right)∙2\sin\left(\theta \right)∙d\theta \sin\left(\theta \right)∙\cos\left(\theta \right)∙\cos\left(\varphi \right)∙\sin\left(\theta +\varphi \right)=2r^{3}\Phi_{E}\int_{0}^{\left(\pi /2\right)}\left(\cos^{2}\left(\theta \right)∙\sin^{2}\left(\theta \right)∙\cos\left(\varphi \right)\right.∙\left(\sin\left(\theta \right)\;\sin\left(\varphi \right)+\cos\left(\theta \right)\cos\left(\varphi \right)\right)d\theta =2r^{3}\Phi_{E}\left(\int_{0}^{\left(\pi /2\right)}\cos^{2}\left(\theta \right)∙\sin^{3}\left(\theta \right)∙\cos\left(\varphi \right)∙\sin\left(\varphi \right)d\theta +\int_{0}^{\left(\pi /2\right)}\cos^{3}\left(\theta \right)∙\sin^{2}\left(\theta \right)∙\cos^{2}\left(\varphi \right)d\theta \right)=2r^{3}\Phi_{E}\left(\int_{0}^{\left(\pi /2\right)}{-\cos}^{2}\left(\theta \right)∙\sin^{2}\left(\theta \right)d\cos\left(\theta \right)∙\cos\left(\varphi \right)∙\sin\left(\varphi \right)+\int_{0}^{\left(\pi /2\right)}\cos^{2}\left(\theta \right)∙\sin^{2}\left(\theta \right)\text{ dsin}\left(\theta \right)∙\cos^{2}\left(\varphi \right)\right)=2r^{3}\;\Phi_{E}\left(-\int_{0}^{\left(\pi /2\right)}\cos^{2}\left(\theta \right)∙\left(1-\cos^{2}\left(\theta \right)\right)d\cos\left(\theta \right)∙\cos\left(\varphi \right)∙\sin\left(\varphi \right)+\int_{0}^{\left(\pi /2\right)}\left(1-\sin^{2}\left(\theta \right)\right)∙\sin^{2}\left(\theta \right)\text{ dsin}\left(\theta \right)∙\cos^{2}\left(\varphi \right)\right)=2r^{3}\;\Phi_{E}\left(\left(-\int_{0}^{\left(\pi /2\right)}\cos^{2}\left(\theta \right)d\cos\left(\theta \right)+\int_{0}^{\left(\pi /2\right)}\cos^{4}\left(\theta \right)d\cos\left(\theta \right)\right)∙\cos\left(\varphi \right)∙\sin\left(\varphi \right)+\left(\int_{0}^{\left(\pi /2\right)}\sin^{2}\left(\theta \right)d\sin\left(\theta \right)-\int_{0}^{\left(\pi /2\right)}\sin^{4}\left(\theta \right)\text{dsin}\left(\theta \right)\right)\cos^{2}\left(\varphi \right)\right)=2r^{3}\Phi_{E}\left(\left(-\frac{1}{3}{\left.\cos^{2}\left(\theta \right)\right|}_{0}^{\left(\pi /2\right)}+\frac{1}{5}\cos{\left.\left(\theta \right)\right|}_{0}^{\left(\pi /2\right)}\right)∙\cos\left(\varphi \right)∙\sin\left(\varphi \right)+\left(\frac{1}{3}{\left.\sin^{3}\left(\theta \right)\right|}_{0}^{\left(\pi /2\right)}+\frac{1}{5}\sin^{5}{\left.\left(\theta \right)\right|}_{0}^{\left(\pi /2\right)}\right)∙\cos^{2}\left(\varphi \right)\right)=2r^{3}\Phi_{E}\left(\frac{2}{15}\cos\left(\varphi \right)∙\sin\left(\varphi \right)+\frac{2}{15}\cos^{2}\left(\varphi \right)\right). \end{array}$$

The denominator is as follows:

(B.4) $$\begin{array}{c} \int_{0}^{\left(\pi /2\right)}2x∙ds\sin\left(\theta \right)∙\Phi_{E}=\Phi E\int_{0}^{\left(\pi /2\right)}2r\sin\left(\theta \right)∙rd\theta ∙\sin\left(\theta \right)=2r^{2}\Phi \text{E}\int_{0}^{\left(\pi /2\right)}\sin^{2}\left(\theta \right)d\theta =2r^{2}\Phi E\int_{0}^{\left(\pi /2\right)}\frac{1-\cos\left(2\theta \right)}{2}d\theta =2r^{2}\Phi E\left[\int_{0}^{\left(\pi /2\right)}\left(\frac{1}{2}d\theta -\frac{1}{2}\cos\left(2\theta \right)d\theta \right)\right]=2r^{2}\Phi E\left(\frac{1}{2}{\left.\theta \right|}_{0}^{\left(\pi /2\right)}-\frac{1}{4}\sin{\left.\left(2\theta \right)\right|}_{0}^{\left(\pi /2\right)}\right)=2r^{2}\Phi E\left[\left(\frac{1}{2}∙\frac{\pi }{2}-\frac{1}{2}∙0\right)-\frac{1}{4}\left(\sin\left(\pi \right)-\sin\left(\theta \right)\right)\right]=r^{2}∙\frac{\pi }{2}\Phi E. \end{array}$$

Therefore, *y*_eff,h_ becomes

(B.5) $$\begin{array}{c} y_{\text{eff},h}=\frac{2r^{3}\left(\left(2/15\right)\cos\left(\varphi \right)∙\sin\left(\varphi \right)+\left(2/15\right)\cos^{2}\left(\varphi \right)\right)}{2r^{2}∙\left(\pi /4\right)}. \end{array}$$

The calculation of the charged particle's beam scatter contribution for effective point calculation of the *y*-axis on the vertical direction is as follows:

(B.6) $$\begin{array}{c} y_{\text{eff},v}=\frac{\int_{0}^{\left(\pi /2\right)}y∙2y∙ds\cos\left(\theta \right)∙\cos\left(\theta \right)∙\cos\left(\varphi \right)∙\sin\left(90-\theta -\varphi \right)∙\Phi E}{\int_{0}^{\left(\pi /2\right)}2y∙ds\cos\left(\theta \right)∙\Phi E}. \end{array}$$

The numerator is as follows:

(B.7) $$\begin{array}{c} \int_{0}^{\left(\pi /2\right)}r\cos\left(\theta \right)\cdot 2r\cos\left(\theta \right)\cdot rd\left(\theta \right)\cdot \cos\left(\theta \right)\cdot \cos\left(\theta \right)\cdot \cos\left(\varphi \right)\cdot \cos\left(\theta +\varphi \right)\cdot \Phi E=2r^{3}\Phi_{E}\int_{0}^{\left(\pi /2\right)}\left(\cos^{4}\left(\theta \right)\cdot \cos\left(\phi \right)\cdot \left(\cos\left(\theta \right)\cos\left(\phi \right)-\sin\left(\theta \right)\sin\left(\phi \right)\right)d\theta \right)=2r^{3}\Phi_{E}\left(\int_{0}^{\left(\pi /2\right)}\cos^{5}\left(\theta \right)\cdot \cos^{2}\left(\phi \right)\cdot d\theta -\int_{0}^{\left(\pi /2\right)}\cos^{4}\left(\theta \right)\cdot \sin\left(\theta \right)\cdot \cos\left(\phi \right)\cdot \sin\left(\phi \right)d\theta \right)=2r^{3}\Phi_{E}\left(\int_{0}^{\left(\pi /2\right)}\cos^{4}\left(\theta \right)d\sin\left(\theta \right)\cdot \cos^{2}\left(\phi \right)-\left(-\int_{0}^{\left(\pi /2\right)}\cos^{4}\left(\theta \right)d\cos\left(\theta \right)\cdot \cos\left(\phi \right)\cdot \sin\left(\phi \right)d\theta \right)\right)=2r^{3}\Phi_{E}\left(\int_{0}^{\left(\pi /2\right)}{\left(1-\sin^{2}\left(\theta \right)\right)}^{2}d\sin\left(\theta \right)\cdot \cos^{2}\left(\varphi \right)+{\left.\frac{1}{5}\cos^{5}\left(\theta \right)\right|}_{0}^{\left(\pi /2\right)}\cos\left(\varphi \right)\cdot \sin\left(\varphi \right)\right)=2r^{3}\Phi_{E}\left(\int_{0}^{\left(\pi /2\right)}\left(1-2\sin^{2}\left(\theta \right)+\sin^{4}\left(\theta \right)\right)d\sin\left(\theta \right)\cdot \cos^{2}\left(\phi \right)+\frac{1}{5}\left(0-1\right)\cdot \cos\left(\phi \right)\cdot \sin\left(\phi \right)\right)=2r^{3}\Phi_{E}\left(\left({\left.\sin\left(\theta \right)\right|}_{0}^{\left(\pi /2\right)}-{\left.\frac{2}{3}\sin^{3}\left(\theta \right)\right|}_{0}^{\left(\pi /2\right)}+{\left.\frac{1}{5}\sin^{5}\left(\theta \right)\right|}_{0}^{\left(\pi /2\right)}\right)\cdot \cos^{2}\left(\varphi \right)-\frac{1}{5}\cos\left(\varphi \right)\cdot \sin\left(\varphi \right)\right)=2r^{3}\Phi_{E}\left(\frac{8}{15}\cos^{2}\left(\varphi \right)=\frac{1}{5}\cos\left(\varphi \right)\cdot \sin\left(\varphi \right)\right). \end{array}$$

The denominator is as follows:

(B.8) $$\begin{array}{c} \int_{0}^{\left(\pi /2\right)}2y\cdot ds\cos\left(\theta \right)\Phi_{E}=\Phi_{E}\int_{0}^{\left(\pi /2\right)}2r\cos\left(\theta \right)\cdot rd\theta \cdot \cos\left(\theta \right)=2r^{2}\Phi_{E}\int_{0}^{\left(\pi /2\right)}\cos^{2}\left(\theta \right)d\theta =2r^{2}\Phi_{E}\int_{0}^{\left(\pi /2\right)}\frac{1+\cos^{2}\left(2\theta \right)}{2}d\theta =2r^{2}\Phi_{E}\left[\int_{0}^{\left(\pi /2\right)}\left(\frac{1}{2}d\theta +\frac{1}{2}\cos\left(2\theta \right)d\theta \right)\right]=2r^{2}\Phi_{E}\left({\left.\frac{1}{2}\theta \right|}_{0}^{\left(\pi /2\right)}+{\left.\frac{1}{4}\sin\left(2\theta \right)\right|}_{0}^{\left(\pi /2\right)}\right)=2r^{2}\Phi_{E}\left[\left(\frac{1}{2}\cdot \frac{\pi }{2}-\frac{1}{2}\cdot 0\right)+\frac{1}{4}\left(\sin\left(\pi \right)-\sin\left(0\right)\right)\right]=2r^{2}\cdot \frac{\pi }{4}\Phi_{E}. \end{array}$$

Therefore, *y*_eff,*v*_ becomes

(B.9) $$\begin{array}{c} y_{\text{eff},v}=\frac{2r^{3}\left(\left(8/15\right)\cos^{2}\left(\phi \right)-\left(1/5\right)\cos\left(\phi \right)\cdot \sin\left(\phi \right)\right)}{2r^{2}\cdot \left(\pi /4\right)}. \end{array}$$
